# Ophthalmic features of HIV associated cryptococcal meningitis in Malawian Adults: an observational study

**DOI:** 10.12688/wellcomeopenres.15067.3

**Published:** 2026-05-07

**Authors:** Jayne P. Ellis, Kate Gaskell, Mary Peirse, Jack Gormley, Newton Kalata, Philip I. Burgess, Patty Mopamboli, Chatonda A. Manda, Christine A. Kiire, Ian Maccormick, Ebbie Gondwe, Síle F. Molloy, Thomas S. Harrison, David G. Lalloo, Robert S. Heyderman

**Affiliations:** 1 Malawi-Liverpool-Wellcome Trust Clinical Research Programme, Blantyre, Malawi; 2 University College London Hospitals NHS Trust, London, UK; 3 Division of Infection and Immunity, University College London, London, UK; 4 London School of Hygiene & Tropical Medicine, London, UK; 5 Department of Eye and Vision Science, University of Liverpool, Liverpool, UK; 6 University of Malawi College of Medicine, Blantyre, Malawi; 7 Oxford Eye Hospital, John Radcliffe Hospital, Oxford, UK; 8 Institute for Infection and Immunity, St George's, University of London, London, UK; 9 Department of Clinical Sciences, Liverpool School of Tropical Medicine, Liverpool, UK

**Keywords:** HIV, cryptococcal meningitis, retinopathy, ophthalmic signs, Africa

## Abstract

**Background:**

Cryptococcal meningitis (CM) is the commonest neurological complication in patients with advanced HIV. Visual disturbance is a frequent presenting symptom. Papilloedema is commonly reported but other ophthalmic findings are not well described.

**Methods:**

We performed an observational study comparing severely immunocompromised HIV-infected patients with and without CM to determine the nature and prevalence of retinal pathology associated with CM. 70 adult patients were enrolled in Blantyre Malawi, 35 with CM and 35 HIV-infected patients without CM.

**Results:**

79% (19/24) of CM patients examined on day one had evidence of retinal abnormalities compared to 17% (6/35) of HIV-infected controls (p < 0.001). In the CM group, retinal whitening was the commonest abnormality (50%), followed by optic disc swelling (29%), haemorrhage (25%) and vascular abnormalities (7%). Retinal whitening was the only abnormality observed in the comparator group (17%). In CM, there was no significant difference between those with and without retinal abnormalities in fungal burden (13,550 cfu/ml vs. 9,150 cfu/ml; p = 0.65), CD4 count (28 cells/μl vs. 76 cells/μl; p = 0.79) or CSF opening pressure (21 cm H20 vs. 27 cm H20; p = 0.5). There was no association between presence/absence of retinal abnormalities and death (40% 10-week mortality vs. 26%; p = 0.6).

**Conclusions:**

Whether the presence of CM retinopathy could be used as a marker of disease severity warrants further investigation. The observed ophthalmic findings provide a descriptive framework for CM retinopathy to be utilised in future CM studies.

**Trial registration:**

ISRCTN ( ISRCTN45035509) 19/06/2012.

## Background

Cryptococcal meningitis (CM) remains a leading cause of death in patients with advanced HIV, accounting for 15% of AIDS-related deaths in 2014. ^1^ Visual disturbances associated with cryptococcal meningitis are common including reduced visual acuity, blurred vision, diplopia and photophobia. In a South African study of patients with HIV-associated CM due to *Cryptococcus neoformans*, 46.5% (40/86) had decreased visual acuity. ^2^ Visual loss was also reported frequently in CM caused by *Cryptococcus gattii,* in 82 immunocompetent patients from Papua New Guinea, visual loss occurred in 52.6% of survivors. ^3^ Visual loss may occur secondary to raised intracranial pressure or be due to direct fungal invasion of the optic nerve, optic chiasm or optic tracts. ^4^ Retinal features associated with CM have not been well-described.

Intracranial hypertension occurs in up to 75% of patients with CM ^5^ and papilloedema is reported to be the commonest retinal finding, ranging between 33% and 48% of patients. ^6^ ^–^ ^9^ In a study of CM patients with catastrophic visual loss, papilloedema was observed in 70% (33/47). ^10^ Other ophthalmic features reported to be associated with CM in case reports include: retinal haemorrhages, ^6^ neuroretinitis ^5^, multi-focal choroiditis, ^11^ optic atrophy, ^12^ vascular tortuosity, ^13^ exudative retinal detachment, ^14^ cloudy vitreous, ^15^ and endophthalmitis. ^16^ To what extent these changes were attributable to CM rather than HIV retinopathy, or other opportunistic infections e.g. CMV retinitis, ^7^ is uncertain.

We performed an observational study comparing severely immunocompromised HIV-infected Malawian adults with and without CM to determine the nature and prevalence of retinal pathology associated with CM. This study was nested within the Advancing Cryptococcal meningitis Treatment for Africa (ACTA) trial. ^17^

## Methods

The ACTA trial was a Phase III multi-centre randomised controlled trial which aimed to identify the optimal treatment of HIV-associated CM in resource limited settings ( ISRCTN45035509). ^17^ HIV-infected adults with CM were randomised to one of five anti-fungal treatment arms and followed up for 10 weeks. The Queen Elizabeth Central Hospital (QECH), Blantyre, Malawi, was the largest recruiting centre. Patients were treated with anti-retroviral therapy (ART) according to national guidelines.

QECH is a large tertiary referral hospital in Southern Malawi. Between March 2014 and July 2015, a subset of consecutive consenting adults from the ACTA trial were recruited into the ophthalmology sub-study. This subset comprised all ACTA patients who were sufficiently stable to comply with a full ophthalmology assessment and able to consent.

Baseline clinical and laboratory data was collected including: duration of ART, CD4 count using case report forms (CRFs); cerebrospinal fluid (CSF) opening pressure was measured using manometers used during lumbar puncture and quantitative cryptococcal culture (QCC) was performed as described previously. ^18^ Dilated indirect ophthalmoscopy using a hand-held ophthalmoscope was performed by a trained ophthalmologist. Visual acuity was tested using the logMAR (logarithm of the minimum angle of resolution) scale. Low vision was defined as a visual acuity less than +0.50 logMAR but equal to or better than +1.3 logMAR.. ^19^ The team aimed to re-examine participants at day 7, week 4 and week 10. Additional examinations however depended upon patient consent, clinical status and availability of an ophthalmologist.

All study participants underwent a detailed ocular examination performed by a trained ophthalmologist experienced in assessing the diagnosing HIV associated eye disease. All retinal findings were recorded on standardized CRFs. If patients presented with symptoms and/or signs consistent with ocular opportunistic infections (OIs) e.g. CMV retinitis or toxoplasmosis, these participants would be referred to the QECH ophthalmology department and/or Medicine Department as appropriate for further assessment and management. Participants presenting with symptoms and/or signs consistent with ocular opportunistic infections were not excluded from the study. Due to the limited diagnostic tests available in our setting, we were unable to conduct serological tests or viral load assays.

To determine whether the observed retinal findings were associated specifically with acute CM as opposed to background advanced HIV disease, from November 2015 to June 2016, 35 HIV-infected otherwise healthy patients with CD4 < 100 cells/μl were recruited by convenience sampling from QECH antiretroviral clinic as a comparator group. Exclusion criteria were: previous episode of CM or current symptoms or signs of meningitis. Baseline clinical data were collected including: CD4 count, ART use and co-morbidities using standardised CRFs. Visual acuity testing using the logMAR scale and dilated indirect ophthalmoscopy using a hand-held ophthalmoscope, was performed at recruitment only.

As this is an observational study, we did not attempt to define retinal pathology as being directly attributable to cryptococcal infection. Rather, our objective was to describe and compare the spectrum of retinal findings observed among adults with advanced HIV disease with cryptococcal meningitis vs. those with advanced HIV disease without cryptococcal meningitis.

*Ethics*: The ACTA trial protocol was approved by the London School of Hygiene and Tropical Medicine Research Ethics Committee (REC) (8854–5) and the University of Malawi, College of Medicine Research Ethics Committee (COMREC) (P.11/11/1149). The sub-study protocol was approved by COMREC (P.11/11/1149). Written informed consent was obtained from all participants.

*Statistical analysis*: Descriptive summaries were used to analyse the observational data as proportions or medians with ranges. We used SAS (version 9.3) to calculate Fisher’s exact or the Mann Whitney tests to compare groups. For the descriptive analysis, ophthalmic abnormalities were divided into 4 groups: (i) retinal whitening which included retinal discolouration, cotton wool spots, white plaques and choroidal lesions (ii) optic disc swelling (iii) haemorrhage and (iv) vascular abnormalities including vascular tortuosity and dilated vessels.

## Results

46 CM patients recruited to the ACTA study were screened for recruitment to the ophthalmology sub-study. Eight patients died before an ophthalmological examination could be performed, one patient was deemed too unwell, one patient was subsequently excluded from the ACTA trial and one patient was lost to follow up. 35 HIV-infected patients without CM were also recruited (see underlying data ^20^).

### Baseline characteristics

The median age was 35 years in both groups (range 29–51 in CM group, 23–62 in HIV-infected comparator group), 71% (25/35) were male in the CM group compared with 51% (18/35) in the HIV-infected comparator group (p = 0.06). All participants had advanced HIV and there was no difference in the median CD4 counts between groups: 28 cells/μl in the CM group vs. 44 cells/μl in the HIV-infected comparator group (p = 0.21). There was no difference in the proportion of patients on ART between groups (16/35, 46% and 14/35, 43%, p = 0.63). There was a nonsignificant difference in median duration on ART between groups: 23 weeks in the CM group compared to 2 weeks in the HIV-infected comparator group (p = 0.09). 11% (4/35) of CM patients reported visual loss at recruitment, but on formal logMAR testing 39% (7/18) had evidence of reduced visual acuity, 2/18 (11%) were blind. Patients with CM were more likely to have reduced visual acuity compared to the HIV-infected comparator group (39% vs. 9%, p = 0.02).

### Ophthalmic signs

79% (19/24) of CM patients examined on day one had evidence of retinal abnormalities compared to 17% (6/35) of the HIV-infected controls (p < 0.001) ( Table 1).

**Table 1.** Prevalence of retinal pathology in Cryptococcal Meningitis (CM) patients.

Time of fundoscopy	Abnormal fundoscopy N (%)	Retinal whitening N (%)	Optic disc swelling N (%)	Retinal haemorrhage N (%)	Vascular abnormality N (%)	Other * N (%)
**CM Week 1**	23/28 (82)	14/28 (50)	8/28 (29)	7/28 (25)	2/28 (7)	2/28 (7)
**CM Week 4–10**	8/13 (61)	6/13 (46)	1/13 (8)	1/13 (8)	1/13 (8)	0/7 (0)
**HIV-infected** **controls**	6/35 (17)	6/35 (17)	0/35 (0)	0/35 (0)	0/35 (0)	0/35 (0)

Footnote: Of the 35 study participants with CM included in the study, 28 participants had dilated indirect ophthalmoscopy and visual acuity testing performed in the first week following enrolment. 7 participants had dilated indirect ophthalmoscopy and visual acuity testing performed at a later time point only, i.e. after 7 days of enrolment. Reasons for examinations being performed beyond day 7 included participant refusal, severe disease states and/or non availability of the study ophthalmologist.

^*^ *Other = cloudy vitreous.*

**Figure 1.**
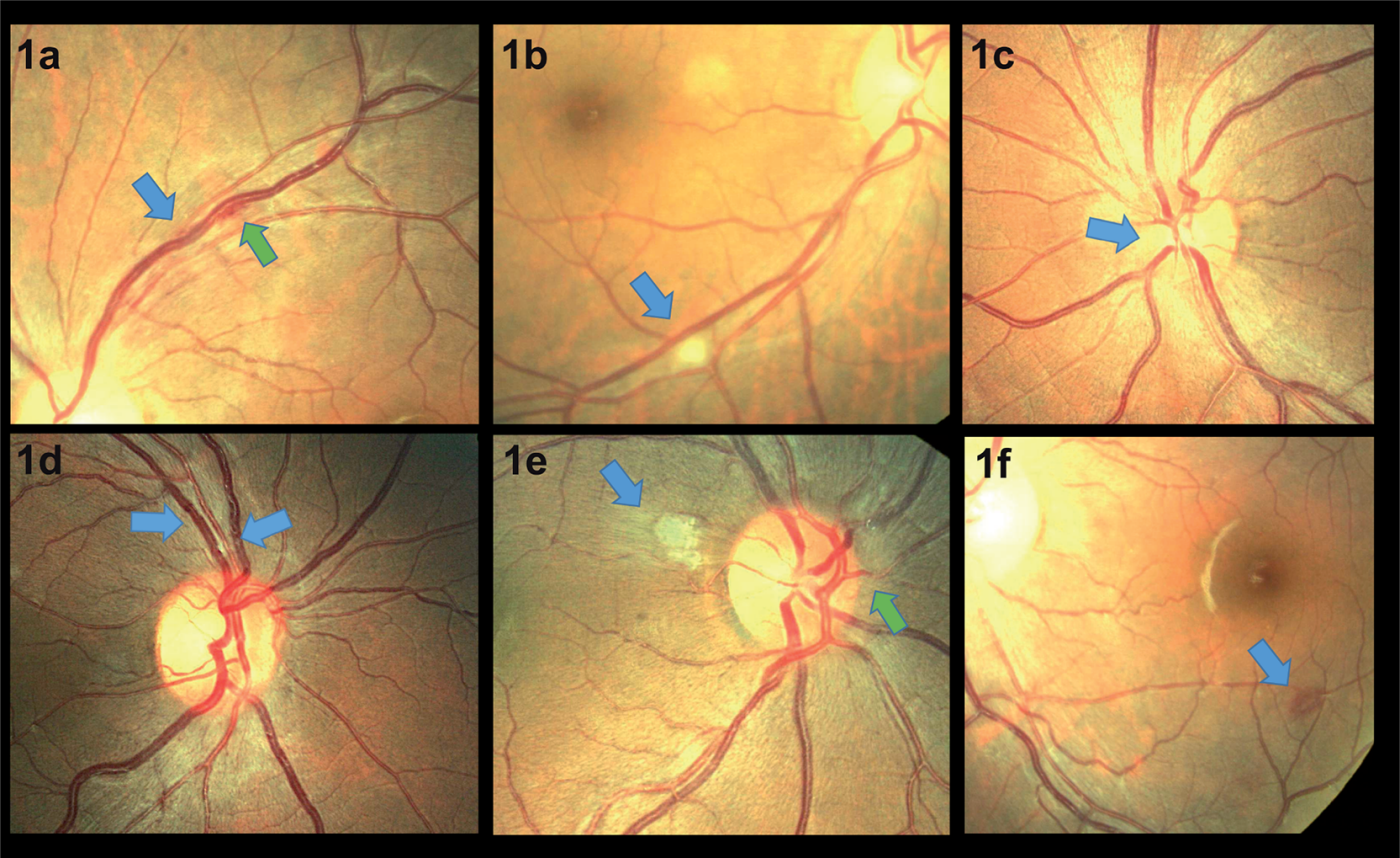
Retinal photographs taken from patients with HIV associated cryptococcal meningitis. **1a** shows localised vascular tortuosity (blue arrow) with associated haemorrhage (green arrow). **1b** shows a cotton wool spot (blue arrow). **1c** shows swelling of the nasal optic disc margin (blue arrow). **1d** shows vascular engorgement and focal tortuosity (blue arrows). **1e** shows atypical retinal whitening (blue arrow) and nasal disc swelling (green arrow). **1f** shows a white centred haemorrhage (blue arrow).

28/35 (80%) of participants underwent dilated indirect ophthalmoscopy and visual acuity testing in the first week. 82% (23/28) of CM patients examined within one week of CM diagnosis had evidence of retinopathy. In this first week, retinal whitening was the commonest abnormality observed (14/28, 50%), followed by optic disc swelling (8/28, 29%), haemorrhage (7/28, 25%), vascular abnormalities (2/28, 7%) and cloudy vitreous (2/28, 7%) ( Table 1, Figure 1). 75% (6/8) of patients with optic disc swelling had raised intracranial pressure (CSF opening pressure > 20 cm H20) and 35% (6/17) of patients with raised intracranial pressure had optic disc swelling. To investigate the relationship between retinopathy and CM disease severity, an association between the following indicators and the presence or absence of retinopathy (observed ≤ one week after CM diagnosis) was explored. There was no significant difference in fungal burden (median QCC: 13,550 cfu/ml vs. 9,150 cfu/ml p = 0.65) or CD4 count (median CD4 count: 28 cells/μl vs. 76 cells/μl, p = 0.79). There was no association between CSF opening pressure at diagnosis (21 cm H20 vs. 27 cm H20; p = 0.5), ART use (12/23, 52% vs. 2/5, 40%; p = 1.0) or baseline haemoglobin (11.2 g/dL vs. 11.1 g/dL; p = 0.4) and the presence of retinal abnormalities. There was no association between presence of retinal abnormalities and death (40% 10-week mortality vs. 26%; p = 0.6).

In contrast to the range of retinal signs seen in the CM patients, retinal whitening was the only abnormality observed in the HIV-infected comparator group (6/35, 17%; Table 1). A subset of CM patients (13/35) had indirect ophthalmoscopy performed between 4–10 weeks after diagnosis and commencement of anti-fungal therapy. Six of these patients had undergone an earlier ophthalmological examination during the first week and seven patients were examined for the first time between week 4–10. Some of these patients (8/13, 61%) had evidence of retinopathy. Retinal whitening remained the most frequent abnormality observed (6/13, 46%). Optic disc swelling, retinal haemorrhages and vascular abnormalities were seen in one patient each (8%). Of the six patients who had previously undergone ophthalmic examination during week one of their CM episode, two patients (33%) had complete resolution of their retinopathy, two patients (33%) had evidence of persistent retinal whitening, one patient (17%) had persistent mild optic disc swelling and one patient (17%) had a normal examination at both time points. Visual acuity was assessed for the two participants with ongoing evidence of retinal whitening at the week 4–10 assessment, using the logMAR scale neither had evidence of persistent functional low vision (defined as a visual acuity less than +0.50 logMAR).

## Discussion

Our observational study highlights the range and relative frequency of ophthalmic abnormalities that are associated with acute CM. Retinal whitening was the most common abnormality observed, followed by optic disc swelling, haemorrhage and vascular abnormalities; these features have each been documented previously in case reports. ^7^ ^,^ ^9^ ^,^ ^10^ Among CM patients who were examined within one week of CM diagnosis, optic disc swelling was observed in 29% which is in line with other CM case series (range 32.5% – 37.5%). ^7^ ^–^ ^9^ Previous CM studies have clearly demonstrated an association between the presence of optic disc swelling and raised ICP. ^21^ Although most patients in our cohort with optic disc swelling had raised intracranial pressure, importantly only a third of patients with raised intracranial pressure had optic disc swelling.

Retinal whitening may be due to oncotic cell swelling in response to reduced perfusion or occlusion caused by microthrombi, ^22^ both of these mechanisms may be important in patients with CM due to the pro-inflammatory, vasculitic nature of the disease. ^23^ ^,^ ^24^ However, retinal whitening was also observed in the HIV-infected comparator group and has previously been described in association with HIV retinopathy. ^25^ Indeed, retinal whitening is observed in a range of retinal vascular disorders including cerebral malaria, retinal arterial occlusion and retinal vein occlusion. ^22^ In some patients retinal whitening may have pre-dated the onset of CM.

The highest prevalence (82%) of retinopathy in CM was observed during the first week after diagnosis. In a subset of patients examined between week 4–10, fewer (61%) had evidence of retinopathy. The resolution of the ophthalmic features associated with CM has not been reported previously ^6^ ^–^ ^8^ ^,^ ^13^ and therefore needs to be investigated in larger cohorts.

Although patients with evidence of CM retinopathy had lower median CD4 counts and higher fungal burdens, our study was underpowered to detect a clear association with these features or indeed death. In a study of 80 HIV-associated CM cases from Rwanda, there was a non-significant trend towards shorter median survival times in patients with cotton wool spots (102 days) compared to those without cotton wool spots (186 days). ^7^ Future larger studies of CM should determine whether the retinal abnormalities associated with CM could be used as a marker of disease severity.

The strength of this study was that it was nested within a randomized controlled trial which provided standardized data collection, diagnosis and treatment. All patients were examined by a trained ophthalmologist and in contrast to other case series, we had a severely immunocompromised HIV-infected comparator group. This study was limited by the relatively small number of participants, many of whom were only examined at a single time point. Our study was therefore underpowered to detect an association between presence of CM retinopathy and clinical outcomes including death. Although the cohort characteristics were similar to the main ACTA trial, ^15^ we have inevitably sampled patients with less severe CM, who were able to tolerate and consent to an ophthalmological assessment. However, we do not anticipate that the patients with more severe CM would have had fewer intraocular abnormalities.

## Conclusion

Optic disc swelling, retinal haemorrhages and vascular abnormalities are commonly observed in patients with acute CM, with the highest prevalence in the first week following diagnosis. Whether the presence of CM retinopathy could be used as a marker of CM severity warrants further investigation. The observed ophthalmic findings provide a descriptive framework for CM retinopathy to be utilised in future CM studies.

## Data availability

Figshare: Ophthalmic features and clinical data of HIV-infected Malawian adults with and without cryptococcal meningitis. https://doi.org/10.6084/m9.figshare.8082275 ^20^

This project contains the following underlying data:

• RDS-SPRN-00282 resubmission.xlsx (Spreadsheet containing demographic, clinical, lab and ophthal data for all participants)

Data are available under the terms of the Creative Commons Zero “No rights reserved” data waiver (CC0 1.0 Public domain dedication).
