# Supplementary material for: Ophthalmic features of HIV associated cryptococcal meningitis in Malawian Adults: an observational study
Source: Wellcome Open Res. 2026 May 7;4:83. Originally published 2019 May 20. [Version 3] doi: 10.12688/wellcomeopenres.15067.3 (PMC13168877; doi:10.12688/wellcomeopenres.15067.3)
Supplement: Peer review history

# Reviewer report: Reviewer response for version 3

**DOI:** 10.21956/wellcomeopenres.29220.r155182

**Published:** 2026-05-12

**Authors:** Ankit Varshney

**Affiliations:** 1 Optometry, Veer Narmad South Gujarat University (Ringgold ID: 29035), Surat, India

Is the work clearly and accurately presented and does it cite the current literature?

- The article is generally well written and easy to follow.
- The background and rationale of the study are clear.
- Relevant previous studies and literature have been cited appropriately.
- However, the manuscript sometimes gives the impression that the retinal findings are directly caused by cryptococcal meningitis, although this cannot be confirmed from an observational study.
- Retinal findings such as retinal whitening may also be due to HIV retinopathy or other opportunistic infections.
- The language throughout the manuscript should be more cautious and should clearly state that these are “associated findings” rather than proven disease-specific findings.

Is the study design appropriate and is the work technically sound?

- The observational study design is appropriate for the research question.
- Including an HIV-positive comparator group without cryptococcal meningitis is a major strength.
- The study being nested within the ACTA trial improves the quality and reliability of the data collection.
- Ophthalmic examinations were performed by trained ophthalmologists, which strengthens the technical quality of the study.
- Overall, the study methods are acceptable for an exploratory descriptive study.

Are sufficient details of methods and analysis provided to allow replication by others?

- Most methods are adequately described.
- Inclusion and exclusion criteria are generally clear.
- Statistical tests used are mentioned appropriately.
- However, some important details still require clarification:
  - Timing of ophthalmic examinations was variable among participants.
  - Only some participants underwent repeat examinations.
  - Visual acuity assessment details are incomplete for all participants.
  - It remains unclear how possible ocular opportunistic infections were differentiated from CM-related findings in resource-limited settings.
- A clearer participant flow diagram and more structured explanation of follow-up assessments would improve reproducibility.

If applicable, is the statistical analysis and its interpretation appropriate?

- Basic statistical methods used in the study are appropriate for observational data.
- Fisher’s exact test and Mann–Whitney tests are suitable for the small sample size.
- However, the study is underpowered due to the relatively small number of participants.
- Several conclusions stating “no association” may simply reflect insufficient statistical power.
- The manuscript should use more cautious wording such as:
  - “No statistically significant association was detected.”
- Confidence intervals were not reported.
- No multivariable analysis was performed.
- Statistical interpretation should therefore be more conservative.

Are all the source data underlying the results available to ensure full reproducibility?

- The authors have made the underlying dataset publicly available through Figshare.
- Clinical, laboratory, demographic, and ophthalmic data are accessible.
- This improves transparency and reproducibility of the study.

Are the conclusions drawn adequately supported by the results?

- The descriptive findings are generally supported by the presented data.
- The study successfully demonstrates that retinal abnormalities are more common in HIV patients with cryptococcal meningitis compared to HIV-positive controls.
- However, some conclusions are slightly stronger than the available evidence supports.
- Due to:
  - small sample size,
  - incomplete follow-up,
  - exclusion of severely ill patients,
  - and inability to confirm causality,
    the findings should be interpreted cautiously.
- The suggestion that retinal findings may serve as markers of disease severity is interesting but remains speculative and requires larger prospective studies.

Some typographical and formatting issues remain:

- “Towhat extent” should read “To what extent”.
- “CMgroup” should read “CM group”.
- “H20” should consistently be written as “H₂O”.
- Extra punctuation appears in:
  “+1.3 logMAR..”
- The manuscript would benefit from clearer organization of participant flow, possibly through a CONSORT-style flow diagram for the ophthalmology substudy.
- Table 1 is informative but could be improved by:
- adding denominators clearly,
- specifying repeated assessments,
- and clarifying overlap between week 1 and week 4–10 participants.

Finally,

 This is a useful and clinically relevant observational study that adds valuable descriptive information regarding ophthalmic findings in HIV-associated cryptococcal meningitis. However, the manuscript would be improved by using more cautious language regarding causality, clarifying methodological details further, and discussing the study limitations more extensively.

Is the work clearly and accurately presented and does it cite the current literature?

Partly

If applicable, is the statistical analysis and its interpretation appropriate?

Partly

Are all the source data underlying the results available to ensure full reproducibility?

Yes

Is the study design appropriate and is the work technically sound?

Yes

Are the conclusions drawn adequately supported by the results?

Partly

Are sufficient details of methods and analysis provided to allow replication by others?

Partly

Reviewer Expertise:

Ophthalmology, Optometry and Vision Science, Neuro-ophthalmology, Retinal Diseases, Ocular Imaging and Diagnostics, Contact Lens and Corneal Research, Public Health Ophthalmology, Community Eye Health and Screening, HIV-related Ocular Manifestations, Clinical Research Methodology, Epidemiology and Biostatistics in Eye Care, Quality of Life Research in Vision Sciences

I confirm that I have read this submission and believe that I have an appropriate level of expertise to confirm that it is of an acceptable scientific standard, however I have significant reservations, as outlined above.

# Reviewer report: Reviewer response for version 2

**DOI:** 10.21956/wellcomeopenres.16952.r36599

**Published:** 2019-11-06

**Authors:** Peter R. Williamson, Seher Anjum

**Affiliations:** 1 Laboratory of Clinical Immunology and Microbiology (LCIM), National Institute of Allergy and Infectious Diseases (NIAID), National Institutes of Health (NIH), Baltimore, MD, USA; 2 Laboratory of Clinical Immunology and Microbiology, National Institute of Health (NIH), Bethesda, MD, USA

Most of the comments from the previous review have been addressed.

Is the work clearly and accurately presented and does it cite the current literature?

Yes

If applicable, is the statistical analysis and its interpretation appropriate?

I cannot comment. A qualified statistician is required.

Are all the source data underlying the results available to ensure full reproducibility?

Yes

Is the study design appropriate and is the work technically sound?

Yes

Are the conclusions drawn adequately supported by the results?

Yes

Are sufficient details of methods and analysis provided to allow replication by others?

Partly

Reviewer Expertise:

Translational mycology (specifically work with Cryptococcus).

We confirm that we have read this submission and believe that we have an appropriate level of expertise to confirm that it is of an acceptable scientific standard.

# Reviewer report: Reviewer response for version 2

**DOI:** 10.21956/wellcomeopenres.16952.r36598

**Published:** 2019-10-24

**Authors:** Héctor Pérez, Claudia Frola

**Affiliations:** 1 Hospital "Juan A. Fernandez", Buenos Aires, Argentina; 2 Infectious Diseases Division, Fernández Hospital, Buenos Aires, Argentina

**From our version 1 report:**

 Background.

 How the retinal pathology attributable to cryptococcus was defined.

***It was not clarified.***

 Methods

 When the duration of antiviral treatment and...

***It was not clarified.***

**From your responses:**

 If patients presented with symptoms and/or signs consistent with ocular opportunistic infections (OIs) e.g. CMV retinitis or toxoplasmosis, these participants would be referred to the QECH ophthalmology department and or Medicine Department as appropriate for further assessment and management. 

***It was not clarified whether they are included or not.***   

 Results

 28/35 (80%) of participants underwent dilated indirect ophthalmoscopy and visual acuity testing in the first week

 Visual acuity was assessed for the two participants with ongoing evidence of retinal whitening at the week 4-10 assessment, using the logMAR scale neither had evidence of persistent functional low vision (defined as a visual acuity less than +0.50 logMAR). 

***Initial sample it should be 35 instead of 28?***

 Conclusions

 We have inevitably sampled patients with less severe CM, who were able to tolerate and consent to an ophthalmological assessment. However, we do not anticipate that the patients with more severe CM would have had fewer intraocular abnormalities.

 Our study was therefore underpowered to detect an association between presence of CM retinopathy and clinical outcomes including death. 

***We agree with the correction.***

Is the work clearly and accurately presented and does it cite the current literature?

Partly

If applicable, is the statistical analysis and its interpretation appropriate?

Yes

Are all the source data underlying the results available to ensure full reproducibility?

Partly

Is the study design appropriate and is the work technically sound?

Yes

Are the conclusions drawn adequately supported by the results?

Partly

Are sufficient details of methods and analysis provided to allow replication by others?

Partly

Reviewer Expertise:

Infectious Diseases Physicians.

We confirm that we have read this submission and believe that we have an appropriate level of expertise to confirm that it is of an acceptable scientific standard, however we have significant reservations, as outlined above.

# Response

**Published:** 2026-04-24

**Authors:** Jayne Ellis

**Affiliations:** Infectious Diseases Institute, Uganda

**Thank you for your review, please find below a point by point response to each of the questions raised:**

 Background.

 How the retinal pathology attributable to cryptococcus was defined.

***It was not clarified.*** We thank the reviewer for this important point and we agree that this was unclear in the earlier version of the manuscript. We would like to clarify that, as this is an observational study, we did not attempt to define retinal pathology as being directly attributable to cryptococcal infection. Rather, our objective was to describe and compare the spectrum of retinal findings observed among adults with advanced HIV disease with cryptococcal meningitis vs. those with advanced HIV disease without cryptococcal meningitis. Accordingly, the study reports associations between retinal pathology and the presence of cryptococcal meningitis, without inferring causality. We have revised the manuscript to make this distinction clearer and to avoid any implication that the retinal findings are definitively caused by cryptococcal infection. This detail has now been added to the methods section. 

 Methods

 When the duration of antiviral treatment and...

***It was not clarified.*** With respect to the duration of anti-retroviral therapy, this is already stated within the 2 ^nd^ paragraph of the results section, under baseline characteristics. To clarify, amongst study participants with CM, the median duration of anti-retroviral therapy at time of study enrolment was 23 weeks. Amongst study participants without CM, the median duration of anti-retroviral therapy at time of study enrolment was 2 weeks. No other antiviral treatments were routinely provided to study participants. **From your responses:**

 If patients presented with symptoms and/or signs consistent with ocular opportunistic infections (OIs) e.g. CMV retinitis or toxoplasmosis, these participants would be referred to the QECH ophthalmology department and or Medicine Department as appropriate for further assessment and management. 

***It was not clarified whether they are included or not.***    Participants presenting with symptoms and/or signs consistent with ocular opportunistic infections were not excluded from the study. This detail has now been added to the methods section.

 Results

 28/35 (80%) of participants underwent dilated indirect ophthalmoscopy and visual acuity testing in the first week

 Visual acuity was assessed for the two participants with ongoing evidence of retinal whitening at the week 4-10 assessment, using the logMAR scale neither had evidence of persistent functional low vision (defined as a visual acuity less than +0.50 logMAR). 

***Initial sample it should be 35 instead of 28? *** Thank you for raising this point for clarification. Of the 35 study participants with CM included in the study, 28 participants had dilated indirect ophthalmoscopy and visual acuity testing performed in the first week following enrolment. 7 participants had dilated indirect ophthalmoscopy and visual acuity testing performed at a later time point, i.e. after 7 days of enrolment. Reasons for examinations being performed beyond day 7 included participant refusal, severe disease states and/or non availability of the study ophthalmologist. This detail has now been added to the results section.   

 Conclusions

 We have inevitably sampled patients with less severe CM, who were able to tolerate and consent to an ophthalmological assessment. However, we do not anticipate that the patients with more severe CM would have had fewer intraocular abnormalities.

 Our study was therefore underpowered to detect an association between presence of CM retinopathy and clinical outcomes including death. 

***We agree with the correction.*** Thank you for this feedback.

# Reviewer report: Reviewer response for version 1

**DOI:** 10.21956/wellcomeopenres.16437.r35940

**Published:** 2019-08-07

**Authors:** Peter R. Williamson, Seher Anjum

**Affiliations:** 1 Laboratory of Clinical Immunology and Microbiology (LCIM), National Institute of Allergy and Infectious Diseases (NIAID), National Institutes of Health (NIH), Baltimore, MD, USA; 2 Laboratory of Clinical Immunology and Microbiology, National Institute of Health (NIH), Bethesda, MD, USA

This is an observational study performed in Malawi comparing retinal findings in HIV patients (with CD4 counts <100) with and without cryptococcal meningitis. The HIV associated CM patient cohort (n=35) is derived from the ACTA study, which was a multi-center RCT done to compare oral and parenteral antifungal therapies to treat CM in resource-limited settings.

- While one of the major aims of this study is contrast between findings attributable to CM versus HIV retinopathy alone, there is no mention of other ocular opportunistic infections being ruled out in those with retinal findings.
- This study may not accurately represent the prevalence/severity of retinal findings in this population since patients who were unstable and couldn’t consent to a complete ophthalmological exam were not included.
- This study would be more informative if the retinal findings could be correlated or defined in terms of the severity of vision loss. For instance, retinal whitening was the most common abnormality found in HIV positive CM patients and persisted in a couple of patients despite antifungals – what degree of visual loss did they have and what does this mean functionally for the patient? It may be better to explain logMAR testing a little more. 
- It is unclear at what time point HIV-positive non-CM patients were being examined. How many months on average had they been on ART when they were examined for the first time? 

Overall,  this is a good descriptive study for retinal findings in HIV positive CM patients. Most cases reported in the literature previously are case reports. However, it is underpowered to study final visual outcomes and to correlate retinal findings with an elevated ICP or morbidity. These caveats should be included under limitations of the study.

Is the work clearly and accurately presented and does it cite the current literature?

Yes

If applicable, is the statistical analysis and its interpretation appropriate?

I cannot comment. A qualified statistician is required.

Are all the source data underlying the results available to ensure full reproducibility?

Yes

Is the study design appropriate and is the work technically sound?

Yes

Are the conclusions drawn adequately supported by the results?

Yes

Are sufficient details of methods and analysis provided to allow replication by others?

Partly

Reviewer Expertise:

Translational mycology (specifically work with Cryptococcus).

We confirm that we have read this submission and believe that we have an appropriate level of expertise to confirm that it is of an acceptable scientific standard, however we have significant reservations, as outlined above.

# Reviewer report: Reviewer response for version 1

**DOI:** 10.21956/wellcomeopenres.16437.r35672

**Published:** 2019-06-24

**Authors:** Héctor Pérez, Claudia Frola

**Affiliations:** 1 Infectious Diseases Division, Fernández Hospital, Buenos Aires, Argentina; 2 Hospital "Juan A. Fernandez", Buenos Aires, Argentina

**Background**

- How the retinal pathology attributable to cryptococcus was defined.

**Methods**

- When the duration of antiviral treatment and the count of lymphocyte sub-populations were considered, it would take into account the time of HIV infection, since the same infection can cause retinopathy associated with it.
- When referring healthy patients with severe immunosuppression <100 CD4, are they considered not having another opportunistic infection, since it only refers to not having cryptococcosis.

**Results**

- The analysis of the results is not clear. Of the thirty-five patients, 28 were analyzed. It is also not clear those who had visual acuity decrease, refers to having made logMAR to eighteen.
- In the table that analyzes the results, it is unclear that thirteen of thirty-five were evaluated between four to ten weeks after diagnosis and the beginning of the antifungal therapy, therefore the findings of retinopathy are inconsistent.

**Conclusions**

- Finally, the findings of retinopathy, co-calculated with the results described, is difficult to consider as a marker of severity in CM, because patients who were more severe were excluded, that is, there could be other associated injuries.

Is the work clearly and accurately presented and does it cite the current literature?

Partly

If applicable, is the statistical analysis and its interpretation appropriate?

Yes

Are all the source data underlying the results available to ensure full reproducibility?

Partly

Is the study design appropriate and is the work technically sound?

Yes

Are the conclusions drawn adequately supported by the results?

Partly

Are sufficient details of methods and analysis provided to allow replication by others?

Partly

Reviewer Expertise:

Infectious Diseases Physicians.

We confirm that we have read this submission and believe that we have an appropriate level of expertise to confirm that it is of an acceptable scientific standard, however we have significant reservations, as outlined above.
